# Supplementary figures and images for: Incoherent Feedforward Regulation via Sox9 and ERK Underpins Mouse Tracheal Cartilage Development
Source: Front Cell Dev Biol. 2020 Oct 22;8:585640. doi: 10.3389/fcell.2020.585640 (PMC7642454; doi:10.3389/fcell.2020.585640)

Figure S1

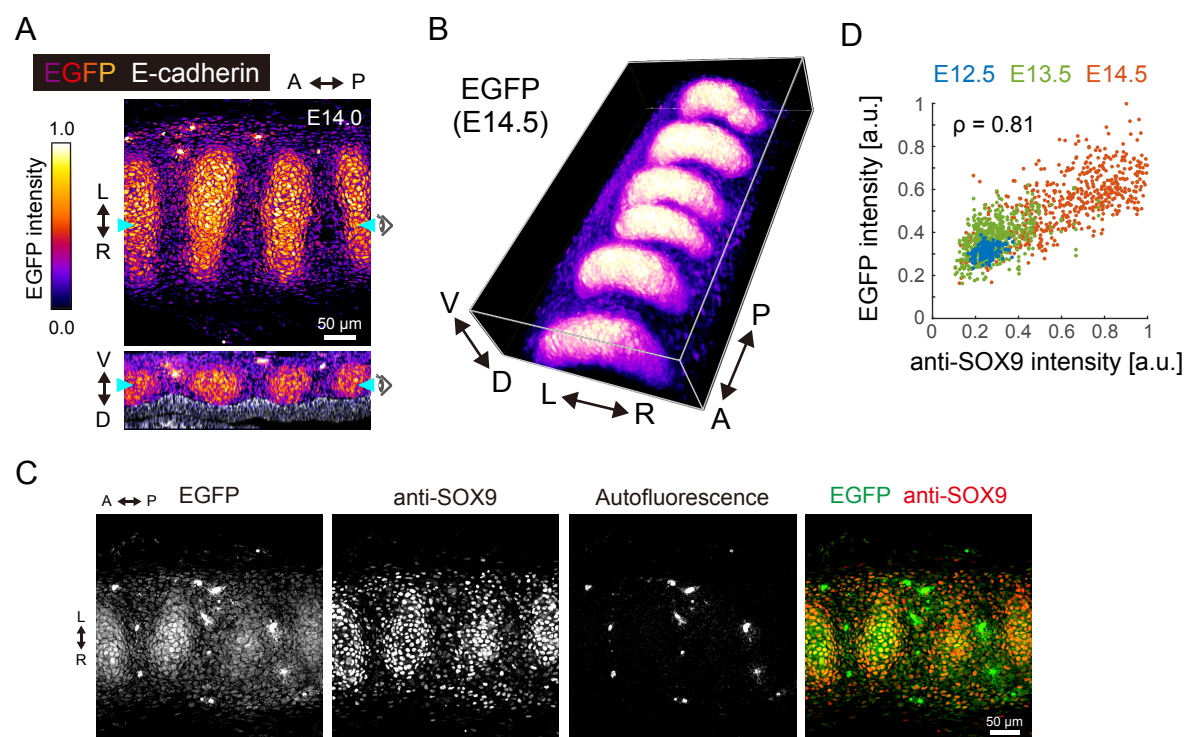

Figure S2

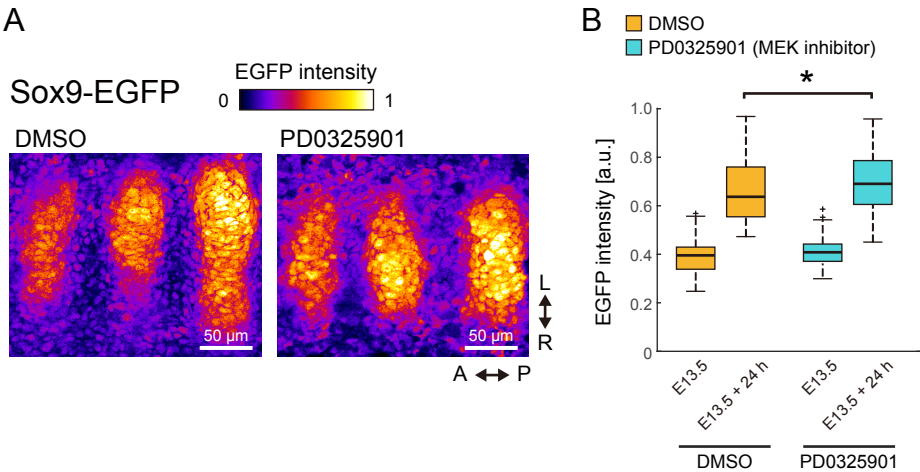

Figure S3

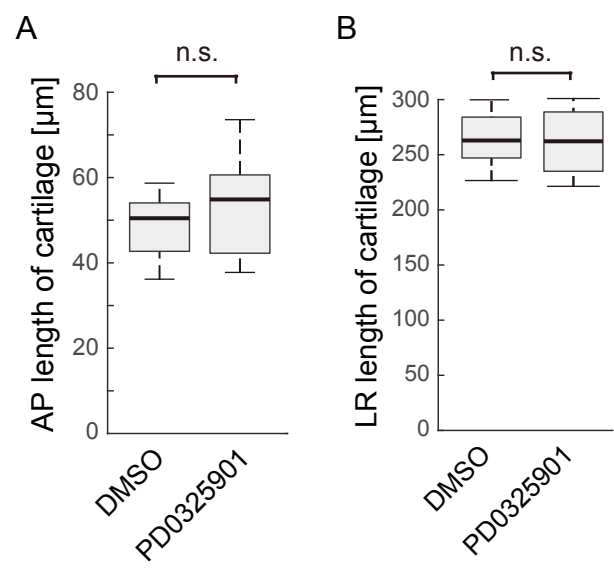

Figure S4

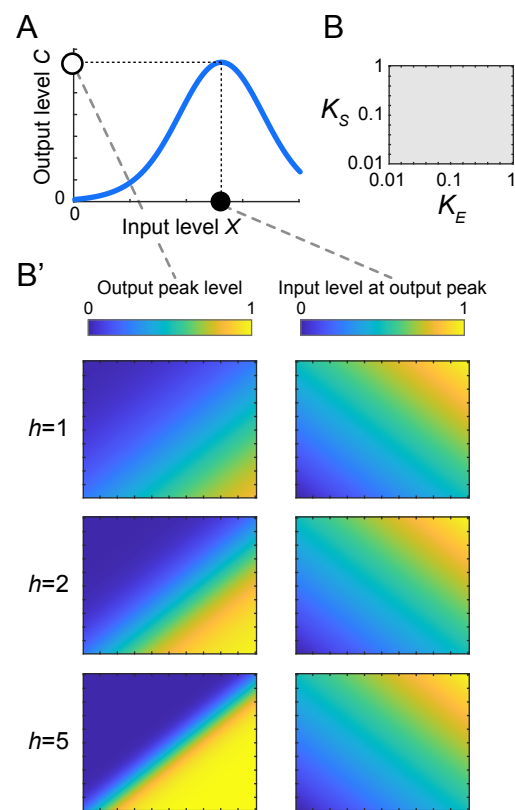

Supplement: Supplementary Figure 1 — Sox9-EGFP profile in the developing murine trachea. (A) A 3D-rendered image of Sox9-EGFP at E14.5, showing the C-shaped tracheal rings on the ventral side. L, left; R, right; A, anterior; P, posterior; V, ventral; D, dorsal. (B) Simultaneous visualization of EGFP (“fire” pseudocolor) and anti-E-cadherin (white) at E14.0. High EGFP expression cell clusters located between the ridges of wavy tracheal epithelium. Scale bar, 50 μm. (C) Simultaneous visualization of EGFP (green) and anti-SOX9 (red) at E14.5. Scale bar, 50 μm. (D) Relationship between EGFP and anti-SOX9 intensity from E12.5 to E14.5. Pearson's linear correlation coefficient: ρ = 0.81. [file Data_Sheet_1.PDF]
